# Five key concepts linking vacancies, structure, and oxygen evolution reaction activity in cobalt-based electrocatalysts

**DOI:** 10.1039/d5cc02438b

**Published:** 2025-07-16

**Authors:** Kenneth Crossley, Thomas J. Schmidt, Emiliana Fabbri

**Affiliations:** a PSI Center for Energy and Environmental Science 5232 Villigen PSI Switzerland emiliana.fabbri@psi.ch; b Institute for Molecular Physical Science, ETH Zürich CH-8093 Zürich Switzerland

## Abstract

Focusing on five key concepts, we review the roles of cation and oxygen vacancies in determining the surface reconstruction pathway, reaction mechanism, and ultimate activity of cobalt-based oxygen evolution reaction (OER) electrocatalysts. Cation and oxygen vacancies can initiate reactant adsorption, facilitating active surface reconstruction, and can switch the dominant mechanism from the adsorbate evolution mechanism (AEM) to the lattice oxygen evolution mechanism (LOEM). However, these effects are facet-dependent. Rigorous oxygen vacancy quantification promises to identify the OER mechanism steering thresholds and unlock the full potential of vacancy engineering. Finally, oxygen vacancy quantification strategies are critically examined to facilitate this goal.

## Abstract 2

Focusing on five key concepts, we review the roles of cation and oxygen vacancies in determining the surface reconstruction pathway, reaction mechanism, and ultimate activity of cobalt-based oxygen evolution reaction (OER) electrocatalysts.

## Graphical abstract

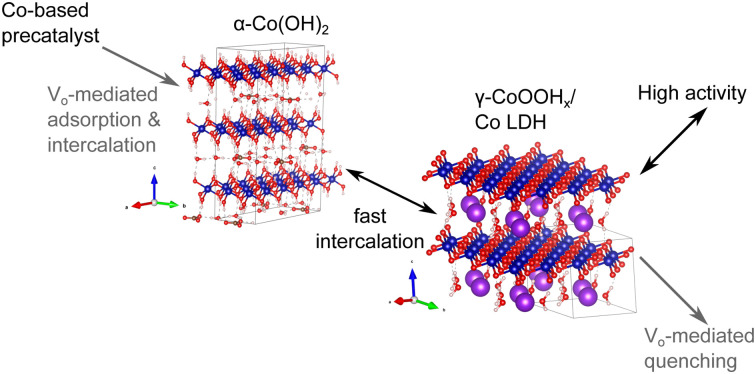

Green hydrogen, produced *via* water electrolysis, is a promising next-generation energy vector. The oxygen evolution reaction (OER) is the rate limiting half-reaction for water electrolysis. OER electrocatalyst cost and performance are important factors impeding global water electrolysis scale up. In an acidic environment, typical industrial OER electrocatalysts are precious metal-based materials (IrO_*x*_, RuO_*x*_). In an alkaline environment, less expensive and more abundant transition metal (hydr)oxides based on Fe, Co, and Ni are becoming competitive. Herein we focus on Co-based electrocatalysts, but the conclusions should generally be applicable to other Fe- and Ni-based materials.

The prevailing aspiration to increase the activity of Co-based (hydr)oxides is to selectively switch the OER mechanism. The prototypical adsorbate evolution mechanism (AEM)—exhibiting concerted proton/electron transfer—has a fundamental activity limit imposed by universal adsorption energy scaling relationships.^1^ Conversely, the lattice oxygen evolution reaction (LOER) breaks these activity limitations *via* non-concerted proton/electron transfer and involves an O–O coupling step between the surface lattice oxygen and adsorbed oxygen species.^2,3^ It is suggested that LOER activation requires a covalent M–O electronic structure with O 2p character near the Fermi level.^4^ The LOER cannot be activated without accompanying dissolution and surface reconstruction under applied potential,^5,6^ but a dynamic equilibrium can be established to balance activity and durability.^7–9^

Multiple LOER mechanisms (LOEMs) have been proposed—including intramolecular nucleophilic attack,^10^ Mars van Krevelen,^11^ and lattice oxygen mechanism (LOM)^4,12,13^—all of which dynamically create/fill oxygen vacancies (V_O_ in the Kröger–Vink notation). The oxide path mechanism (OPM) has also been proposed to occur *via* O–O radical coupling between adjacent metal adsorbate sites when the metal–metal distance is short enough, although it is more common in acidic conditions.^14^ The AEM, LOEM, and OPM are depicted in Scheme 1. Presently, it remains difficult to experimentally differentiate between, and selectively activate, these non-AEM mechanisms (see Concept 3).

**Scheme 1:**
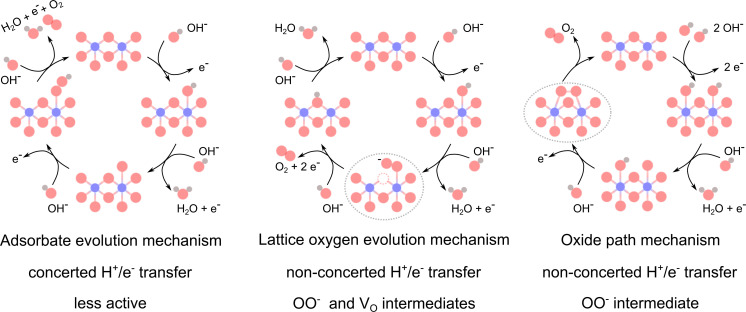
Proposed mechanisms for the alkaline OER. Co atoms are shown in blue, O in red, H in grey, and oxygen vacancies (V_O_) in dashed red circles. The characteristic steps with superoxide OO^−^ intermediates are indicated with grey dashed ellipses. Scheme inspired by Rong *et al.*^14^

In this review, we highlight recent works which indicate key variables to control surface reconstruction pathways, active site geometry, and selective mechanism activation. We structure these works around five key concepts with a view towards fundamental/mechanistic OER understanding.

## 1 Vacancies do more than dangle bonds

Recent works on amorphous OER catalysts/amorphous surface layers often attribute increased activity to flexible structure, increased surface area, or dangling bonds.^15–17^ While these geometric factors certainly contribute, the effects of oxygen vacancies are far more nuanced, can be disentangled far more rigorously, and do not universally improve catalytic activity.

The primary effects of oxygen vacancies are modulating the adsorption site density and free energies of adsorption. As clearly investigated and summarized by Tao *et al.*, introducing surface oxygen vacancies to p-type semiconductors shifts the hybridized O 2p–M 3d antibonding states closer to the Fermi level and increases the metal site adsorption energy of O intermediates on pristine precatalysts.^18^ Note that density functional theory (DFT) calculations of adsorption energies on the actual reconstructed active surfaces remain elusive. Moreover, high bulk oxygen vacancy concentrations can down-shift the O 2p band and reduce the catalytic activity.^19^ Particularly in double perovskites such as PrBaCo_2_O_6−*δ*_, high oxygen deficiency (*δ* ≈ 0.5) can lead to bulk ordering of oxygen vacancies, decreasing conductivity, and a detrimental Co^3+^ high-to-low spin state transition.^20^ Cheng *et al.* examined a La_1−*x*_Sr_*x*_CoO_3−*δ*_ series, a LaMO_3−*δ*_ series (M = Cr, Mn, Fe, Co, Ni), Ba_0.5_Sr_0_.5Co_0.8_Fe_0.2_O_3−*δ*_ and PrBaCo_2_O_6−*δ*_ perovskites and found *δ* > 0.2 to be desirable for OER applications.^21^

Intuitively, the OH^−^ and H_2_O species can also adsorb to oxygen vacancies. Oxygen vacancies and OH^−^ adsorption have been linked to initiating surface reconstruction in many Co-based electrocatalysts including CoSn(OH)_6_,^22^ CoMoO_4_/CoWO_4_/Co_2_VO_4_,^23^ Co(OH)_2_,^24^ and Co_3_O_4_.^25^ The roles of these adsorption sites and species in surface reconstruction are examined in more depth in Concept 4.

Cation vacancies can also positively affect the OER activity. Chen *et al.* used Ar plasma treatment on Co_0.9_Fe_0.1_Sn(OH)_6−*δ*_ to selectively form Sn and O vacancies.^26^ The cation vacancy selectivity was achieved by a relative difference in M–OH bond strengths between the cations. Their defective material showed increased hydrophilicity, a Co/Fe-rich amorphous surface layer, and enhanced electrochemical activity compared to the untreated parent material. Their DFT results suggest that the selective Sn–O vacancies decreased the coordination number and free energy of O adsorption (Δ*G*^0^(O*) = 3.69 *vs.* 2.49 eV) at the Co sites, leading to a lower Tafel slope (77 *vs.* 42 mV dec^−1^) and a shift in the rate determining step compared to the pristine material. Zhang *et al.* have also demonstrated beneficial Co vacancies in Co_3−*x*_O_4_ derived from glycerolatocobalt(ii) pyrolysis.^27,28^ The Co vacancies were confirmed combining X-ray diffraction (XRD), transmission electron microscopy (TEM) energy dispersive spectroscopy, and positron annihilation lifetime spectroscopy measurements. Their DTF results suggest that the Co vacancies introduce a high density of unoccupied states above the Fermi level and increase electron delocalization. These combined effects yielded a turnover frequency an order of magnitude higher than that of pristine Co_3_O_4_.

The extent of dissolution-derived Co vacancies during alkaline OER depends on the precatalyst structure, the presence of other metal ions, the pH, and the applied potential. Moysiadou and Hu used *operando* electrochemical quartz crystal microbalance and inductively coupled plasma optical emission spectroscopy (ICP-OES) to determine the dissolution rates of amorphous electrodeposited CoO_*x*_, CoFeO_*x*_, and CoFeNiO_*x*_ in 1 M KOH at 1.58 V *vs.* RHE (reversible hydrogen electrode).^29^ The CoFeNiO_*x*_ mass remained constant, but CoFeO_*x*_ and CoO_*x*_ showed 20–30% mass loss in the first 6 h before equilibrating with trace Fe adsorption from the electrolyte. In contrast, crystalline Co_3_O_4_ and CoFe_2_O_4_ (111) epitaxial thin films remain stable except for sub-nanometer dissolution and reconstruction at the surface.^30,31^ Lopez *et al.* coupled rotating disk electrode and inductively coupled plasma mass spectrometry (ICP-MS) characterizations to evaluate *operando* dissolution in La_1−*x*_Sr_*x*_CoO_3_ nanoparticles.^32^ La_1−*x*_Sr_*x*_CoO_3_ showed increasing Co dissolution with increasing Sr content, but negligible Co dissolution above 1.5 V *vs.* RHE in pristine 0.1 M KOH and at all tested potentials when 1 ppm Fe was introduced into the electrolyte. Conversely, CoOOH on Pt showed an increasing rate of Co dissolution in the Fe contaminated electrolyte above 1.4 V *vs.* RHE and a 3× lower stability factor. Mn incorporation has also been shown to help stabilize Co dissolution.^33,34^ Overall, it is becoming clear that a dynamic equilibrium must be achieved between the catalyst bulk, reconstructed surface, and transition metal ions in the double layer (see Concept 4).^7,8^

Vacancies created by introducing sacrificial cations in the structure can also selectively influence the active site population, stability, and geometry. Menezes *et al.* demonstrated that selective Zn etching from ZnCo_2_O_4_ preferentially exposes octahedral Co sites at the reconstructed interface.^35^ Ca- and Fe-dominated dissolution in brownmillerite-type Ca_2_FeCoO_5_ drives the transformation to an amorphous CoOOH structure which is stable for at least 4 weeks under OER conditions.^36^ Wei *et al.* investigated a La_0.3_Sr_0.7_Co_1−*x*_Al_*x*_O_3−*δ*_ material series and concluded that Al^3+^ dissolution initiated surface reconstruction *via* oxygen vacancy formation, but that the equilibrium state down-shifted the O 2p band and prevented continued bulk reconstruction.^37^ Liu *et al.* found that this dynamic equilibrium of Al^3+^ dissolution/Al(OH)_*n*_^−^ adsorption was responsible for improved activity, stability, and Cl^−^ repulsion in CoFeAl layered double hydroxides (LDHs) exhibiting high sea water electrolysis performance.^9^

## 2 The effect of surface oxygen vacancies is facet-dependent

Using NaBH_4_ reduction of Co_3_O_4_ nanoparticles, Chen *et al.* introduced surface oxygen vacancies to cubic ((001) facets) and truncated octahedron ((111) majority, (001) minority facets) geometries.^38^ Both types of defective particles outperformed their pristine parent materials, yet, the defective cubic material showed an order of magnitude higher activity and 2–4× greater pH dependence than the pristine cubic and defective octahedral particles. Their DFT results indicate that the upshift of the O 2p band center for the (001) facet reduced the Co 3d and O 2p interband center gap and increased the overlapping O and Co density of unoccupied states just above the Fermi level. There was little change for the (111) facet. Along with *operando* Raman spectroscopy and tetramethylammonium (TMA) oxygen radical quenching measurements, these findings point to the selective activation of the LOEM on oxygen deficient Co_3_O_4_ (001). This AEM to LOEM shift and the relation to surface reconstruction is further discussed in Concepts 3 and 4. Davis *et al.*^39^ and Wei *et al.*^40^ have shown that the extent of Co_3_O_4_ surface reconstruction is also facet-dependent.

Considering other Co_3_O_4_ facets, Shojaee *et al.*'s DFT calculations reveal that it is easier to form oxygen vacancies on the Co_3_O_4_ (100) facet than the (110) facet.^41^ Similarly, the Co_3_O_4_ (220) facet has been calculated to have an even lower oxygen vacancy formation energy,^42^ which could be advantageous to exploit for OER.

This facet dependence is not limited to Co_3_O_4_. Co(OH)_2_ derived β-CoOOH hexagonal nanosheets have been shown to preferentially drive OER on the lateral facets, with the larger area (0001) basal planes being mainly inactive.^43,44^ Similar lateral facet-dominant activity and facet-dependent reconstruction has also been confirmed for NiOOH nanosheet OER catalysts.^45^ Introducing oxygen vacancies to the lateral (101̄0) facets slightly reduces the rate determining potential barrier (−0.05 V), whereas a reduction of −0.4 V is seen for the (0001) and (11̄00) facets.^44^ This large barrier reduction indicates that a combined doping/dissolution plus oxygen vacancies engineering strategy may be sufficient to activate the CoOOH/NiOOH basal (0001) plane for OER.

## 3 The OER mechanism can be controlled *via* oxygen vacancy concentrations

Differentiating between the multiple OER mechanisms remains a challenge. The LOEM and OPM show pH-dependent activity, the order of which should be determined with a fixed overpotential to avoid mixed kinetic–thermodynamic reaction order changes. The intermediate O_2_^−^ radical is present in both the LOEM and OPM, which can be detected *via* TMA quenching,^12,38^ the ^18^O/^16^O kinetic isotope effect,^46^ or Fourier transform infrared spectroscopy.^38,47,48^ Labeling catalysts with ^18^O isotopes along with online mass spectrometry^10,19,49^ or *operando* Raman spectroscopy^50^ can help differentiate between the AEM, LOEM, and OPM. *Operando* application of one or more of these techniques aids in the mechanistic interpretation of pH-dependent activity.

**Fig. 1:**
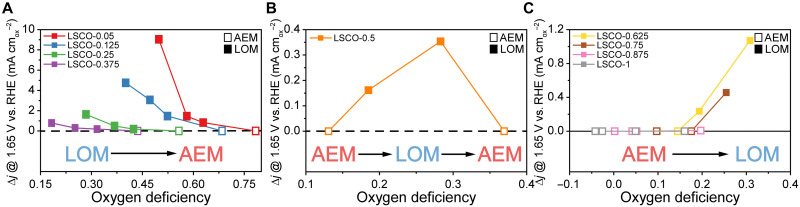
Patterns in OER mechanism shifts for La_*x*_Sr_1−*x*_CoO_3−*δ*_ (LSCO-*δ*). (A) Increasing oxygen deficiency in the high Sr regime deactivates the LOEM. (B) Increasing oxygen deficiency in the mid Sr/La regime activates and then deactivates the LOEM. (C) Increasing oxygen deficiency in the La majority regime activates the LOEM, but the high La range always follows the AEM. Δ*j* is the normalized current density difference from the initial to the maximum activity cycle. For each material, moving left to right increments the ball milling time (0, 2, 4, 6 h). Reproduced from Lu *et al.* (CC-BY-NC 4.0).^19^

To decouple the multiple effects of introducing oxygen vacancies by aliovalent cation doping, Lu *et al.* employed ball milling for incremented times to increase the oxygen vacancy concentrations in a series of La_*x*_Sr_1−*x*_CoO_3−*δ*_ (LSCO-*δ*) materials with fixed cation stoichiometries.^19^ The activity change of this doping + ball milled oxygen vacancies series is reproduced in Fig. 1. The authors clearly demonstrate that increasing the oxygen deficiency can either deactivate the LOEM (high Sr content, Fig. 1(A)) or activate the LOEM/LOM (high La content, Fig. 1(B) and (C)) during CV cycling depending on the Co 3d and O 2p band alignments. In the case of deactivation, the activity-limiting “lockup effect” reflects decreasing participation of the possible OER active sites as the material's cation reducibility limit is approached with increasing oxygen deficiency.^6,19^ Beyond pH-dependent activity and DFT calculations, their Raman spectroscopy results also demonstrate switchable surface reconstruction pathways associated with CoOOH formation.

LOER activation does not appear to be dependent on the method of oxygen vacancy introduction. When Chen *et al.* introduced surface oxygen vacancies to their cubic Co_3_O_4_ nanoparticles *via* NaBH_4_ reduction, they detected increased OER activity and pH dependence compared to the pristine material which followed the AEM.^38^ Their *operando* Raman measurements showed peaks at 1095 and 1125 cm^−1^, which correspond to Co–O–O–Co vibrational modes and indicate an O_2_^−^ intermediate formed by a LOEM or OPM. In addition, the activity decreased with TMA–O_2_^−^ quenching. These results indicate the selective activation of an O_2_^−^ radical-producing mechanism on Co_3_O_4_ (001) facets. Similarly, Zhou *et al.* created surface oxygen vacancies in Co_3_O_4_*via* chemical reduction with increasing concentrations of NaBH_4_.^10^ Their electrochemical, kinetic isotope effect, *operando* Raman spectroscopy, and online mass spectrometry characterizations indicate that the oxygen vacancies activated the LOER and shifted the mechanism to intramolecular nucleophilic attack (metal-adsorbed O attacking adjacent lattice O). However, as discussed in Concept 4, high oxygen vacancy densities can also introduce quenching mechanisms that limit the OER activity.

## 4 Vacancies can steer the surface reconstruction pathway and ultimate catalyst activity

Universally, metal (hydr)oxide catalysts have thermodynamically unstable interfaces under OER conditions and will self-reconstruct with varying degrees of dissolution.^5^ Fabbri *et al.* first applied *operando* X-ray absorption spectroscopy (XAS) to observe the surface reconstruction of oxygen deficient Ba_0.5_Sr_0.5_Co_0.8_Fe_0.2_O_3−*δ*_ to form a (CoFe)O_*x*_(OH)_*y*_ layer.^7^ Using *operando* and *ex situ* freeze-quenched XAS, Bergmann *et al.* demonstrated that CoO (in rock salt and wurtzite structure), Co_3_O_4_, and CoOOH all reconstruct to a principally octahedrally-coordinated 3D cross-linked CoO_*x*_(OH)_*y*_ surface layer under neutral and alkaline OER conditions.^51^ They identified reducible di-μ-oxo-bridged Co^3+^ ions as the reconstructed active site common to all these materials. Despite a common meta-stable active layer, a wide range of activities are observed for Co-based OER electrocatalysts. The reconstruction pathway is key to determining the final activity.

There is mounting evidence that Co (hydr)oxide surfaces with oxygen vacancies take different reconstruction pathways compared with their pristine counterparts. Using *operando* XAS and X-ray photoelectron spectroscopy (XPS), Xiao *et al.* demonstrated that Ar plasma-derived oxygen vacancies increase the adsorption of OH groups at lower potentials compared to defect-free Co_3_O_4_.^25^ Their defective Co_3_O_4_ displayed a lower charge transfer resistance above 1.15 V *vs.* RHE and a faster rate of oxidation/deprotonation before 1.45 V *vs.* RHE. Moreover, Alex *et al.* have shown that crystalline Co_3_O_4_ with oxygen vacancies can have higher reconstructed intrinsic activity and outperform nanocrystalline/amorphous Co_3_O_4_.^52^ Despite minimal long range order and 4.7× more surface area, the nanocrystalline catalyst was reported to have fewer surface oxygen vacancies (based on the troublesome O 1s XPS adsorbed OH peak, see Concept 5) and a lagging Tafel slope of 153 mV dec^−1^. Conversely, Liu *et al.* reported that amorphous Co(OH)_2_ nanocages with abundant oxygen vacancies (detected by electron paramagnetic resonance) outperform crystalline Co_3_O_4_ and demonstrate faster oxidation/reduction at lower potentials.^24^ Overall, it is becoming clear that (oxy)hydroxide species adsorbed on or near oxygen vacancies can reconstruct more easily than on fully ordered facets.

Xiao *et al.* insightfully utilized *operando* Raman spectroscopy to observe the surface reconstruction of pristine and oxygen deficient CoMoO_4_, CoWO_4_, and Co_2_VO_4_.^23^ They showed that oxygen vacancies accelerate Mo/W/V dissolution, thus exposing more oxygen vacancies and Co sites. Oxygen vacancy-adsorbed OH and H_2_O then formed hydrated amorphous Co(OH)_2_ within 5 min at 1.15 V *vs.* RHE. Within 5 min at 1.2 V *vs.* RHE, the intercalated amorphous Co(OH)_2_ converted to CoOOH. Intriguingly, when the defective samples were soaked at open circuit potential in 1 M KOH for 60 min, the oxygen vacancies were filled, and CoO_*x*_ and crystalline Co(OH)_2_ formed. Neither the pristine materials nor the 60 min soaked samples displayed the Raman shifts associated with either water adsorption or CoOOH formation at 1.2 V *vs.* RHE. Both had lower OER activities than the fresh defective sample. Such a defect adsorption/H_2_O intercalation reconstruction mechanism at least partially explains why CoOOH reconstructed from boride/phosphide/sulfide precatalysts often outperforms directly synthesized CoOOH and Co_3_O_4_.^53–58^ Taken together, these results suggest that H_2_O adsorption and intercalation into the reconstructed Co(OH)_2_/CoOOH with an applied potential are necessary for high activity.

Akin to the α-/β-Ni(OH)_2_ system, Leng *et al.*^59^ and Sanchis-Gual *et al.*^60^ examined β-Co(OH)_2_, and anion/H_2_O intercalated α-Co(OH)_2_ as OER precatalysts. They found that α-Co(OH)_2_ exhibited the most reconstruction. Leng *et al.*^59^ and Dionigi *et al.*^61^ further confirmed that β-/α-Co(OH)_2_ selectively reconstruct to β-/γ-CoOOH, with γ-CoOOH exhibiting the higher OER activity. Recently, Wang *et al.* stabilized γ-CoOOH in an alkaline electrolyzer and demonstrated 1 at 1.78 V.^62^ These results further confirm that the reconstructed CoOOH layers OER activity depends on the reconstruction pathway.

**Scheme 2:**
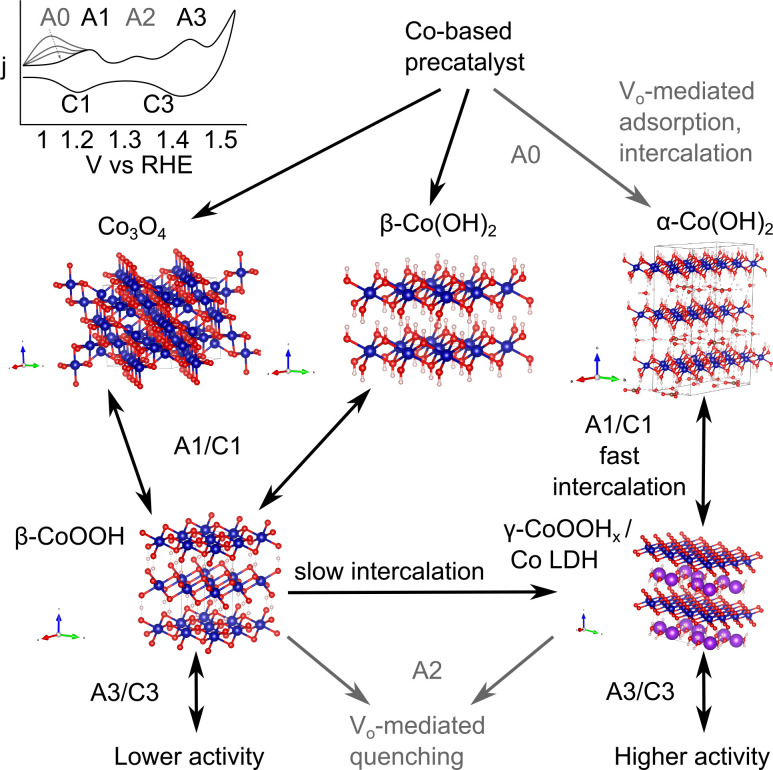
The surface reconstruction pathways followed by Co-based OER catalysts. Oxygen is shown in red, hydrogen in cream, and cobalt in blue. α-Co(OH)_2_ is intercalated by water and anions (*i.e.* CO_3_^2−^ with C shown in brown), whereas γ-CoOOH_*x*_ is intercalated by cations (*i.e.* K^+^ shown in purple).^61^ Co_3_O_4_,^64^ β-Co(OH)_2_,^65^ β-CoOOH_*x*_^66^ data accessed *via* the Crystallography Open Database. Note that the more active intermediate form of γ-CoOOH_*x*_*vs.* a Co layered double hydroxide (LDH) structure is not yet fully clear and may be material specific.

K. Fan *et al.* have proposed different surface reconstruction pathways for CoOOH-like surfaces.^63^ As shown in Scheme 2, after oxidation of Co^2+^ species to β-CoOOH species, a bifurcation occurs. A slow deprotonation and water intercalation step leads to γ-CoOOH_*x*_, which can then be further oxidized to the OER active site or quenched by dense oxygen vacancy concentrations. A faster deprotonation pathway to β-CoO_2_ produces sites with lower intrinsic activity.^63^ Based on the works previously discussed in this section, we propose an additional pathway mediated by oxygen vacancies and intercalated electrolyte species which proceeds directly to γ-CoOOH_*x*_ or a Co LDH structure.

In regions with high oxygen vacancy density, there is evidence suggesting an additional reconstruction pathway which quenches the oxidized Co active sites. For example, Zhou *et al.*'s highest oxygen vacancy concentration sample (1 M NaBH_4_ treatment) displayed two distinct electronic environments and a lower turnover frequency than the intermediate vacancy concentration sample.^10^ Fan *et al.* proposed the following quenching reaction for high oxygen vacancy density environments which increases the number of reconstructed Co^2+^ spectator sites.^63^

1 Co^4+^ + Co–V_O_ + H_2_O → Co^2+^ + Co–O + 2H^+^

We have included the Co^4+^ species by convention, but the quenched species could also be a Co^3+^ coordinated with a oxygen ligand electron hole in covalent systems. The authors proposed that amorphous materials are more prone to this quenching pathway, which may contribute to an increasing degree of crystallinity in amorphous catalysts during/post OER. However, such a chemical reaction does not explain the third anodic peak (A2 in Scheme 2) observed between the typical Co^2+/3+^ (A1) and Co^3+/4+^ (A3) peaks in some amorphous or high oxygen vacancy materials.^10,24^ An additional electrochemical reaction which could account for this activity quenching in high oxygen vacancy density regions below the Co^3+/4+^ oxidation potential follows.2
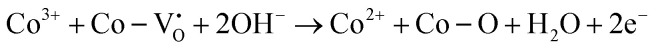


Regardless of the exact species/mechanism(s), note that such quenching reactions are dynamic competing processes during surface reconstruction, whereas the lockup effect originates from a fundamental charge conservation limit.

## 5 Oxygen vacancy quantification is complex and demands user knowledge

To determine exact oxygen vacancy thresholds to steer the reaction mechanism and surface reconstruction pathways, we need robust quantification strategies to be widely implemented. However, many of these techniques are misused or misunderstood in the interdisciplinary electrocatalysis literature. The advantages and disadvantages of various oxygen vacancy quantification techniques are summarized in Table 1.

**Table 1:** Summary of the advantages and disadvantages of oxygen vacancy quantification methods. Accuracy in this context is a qualitative metric balancing precision (typical reproducible significant figures) and specificity (target species sensitivity, technique limitations)

Technique	Accuracy	Advantages	Disadvantages
XPS O 1s 531–532 eV	Low	None	Measures hydroxyls, not V_O_
XPS cation oxidation + normalized lattice O 1s + binding energy	Medium	Quantitative treatment possible	Material specific choices/knowledge requirements
H_2_O_2_/probe redox + colorimetric detection	Medium	Probes surface V_O_ in a liquid environment	Requires standards, unproven hydroxide catalyst applicability
EPR spectroscopy	Medium	Directly detects single electrons trapped at V_O_	Full quantification requires valence state/magnetic characterization
X-ray diffraction	Medium	Low sample masses possible	Less sensitive to light elements
Neutron diffraction	High	High sensitivity to light elements	Large masses, specialized facilities
X-ray absorption spectroscopy	Medium high	Sensitive to bulk V_O_	Less sensitive to surface V_O_
Thermogravimetry	Medium high	Directly measures mass loss for thermal/gaseous V_O_ creation	Gas species detection (ICP-MS/OES) required for definitive attribution
Iodometric titration	Very high	Standard O quantification	Difficult back calculation for multiple, multi-valent transition metals

### 5.1 Near surface techniques

The recent trend of using the fitting area ratio of XPS O 1s peaks at approximately 530 eV (lattice O) and 531–532 eV (often misattributed to oxygen vacancies) to quantify oxygen vacancy densities is fundamentally incorrect.^67,68^ In the case of the O 1s region, XPS measures electrons ejected from core atomic orbitals; thus, there cannot be a signal from a chemical species defined by a missing O atom. Using *in situ* XPS, Yamamoto *et al.* have clearly demonstrated that the 531–532 eV features are characteristic of OH groups formed by the dissociation of water on metal or metal oxide surfaces.^69^ This process will spontaneously fill surface oxygen vacancies upon contact with ambient water vapor or electrolyte.^2,39,67^ Although there is a correlation with this surface hydroxyl spectral feature and surface reconstruction/subsequent OER activity,^10,25^ using the hydroxyl XPS O 1s peak method is not a robust measure of oxygen vacancy concentrations because there are multiple surface hydroxyl adsorption sites^70^ and spectral overlaps with contaminants (*i.e.* Na/K/Ca/Mg chlorides {Auger}, (bi)carbonates).^70,71^

Despite this recent trend, it is often possible to quantify surface oxygen vacancies in a more precise manner using XPS. Wang *et al.* advocate a three-fold quantification strategy using cation valence state peak ratios, lattice O 1s peak ratios, and binding energy shifts.^68^ For the cation valence state peak ratio approach, one must account for/exclude cation protonation and anion redox. When using the lattice O 1s peak approach, one must first normalize the spectra to the baseline or a redox-inactive cation peak intensity. Then the normalized lattice O peak (*A*(≈530 eV)) fitting area can be compared to a fully oxidized surface using the following equation.^68^3
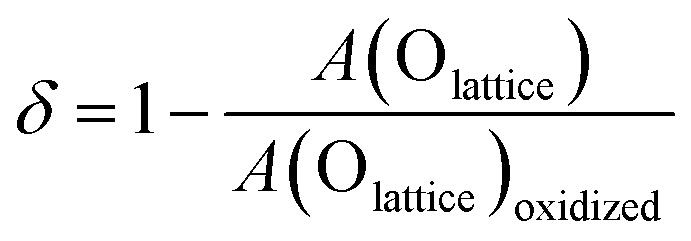


If sample charging, band bending, and space-charge layer effects are minimal, the binding energy shift can be a third strategy to quantify oxygen vacancy concentrations. One must understand the sample to select the appropriate method with XPS.

**Fig. 2:**
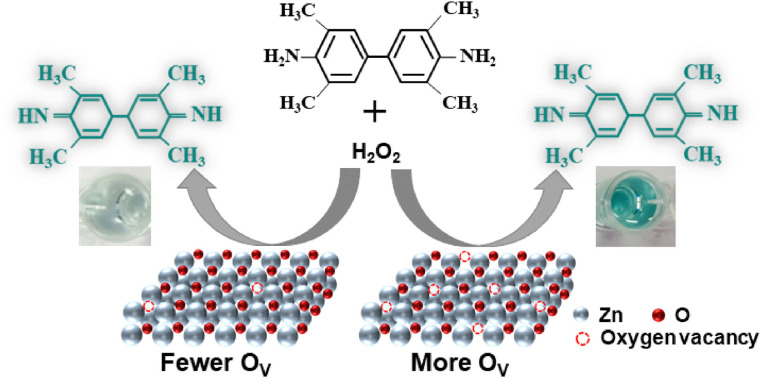
A schematic view of the colorimetric surface oxygen vacancy quantification method demonstrated by Li *et al.*^72^ Reprinted with permission from Elsevier, copyright 2025.

Alternatively, Li *et al.* have developed a rapid, inexpensive, and quantitative colorimetric method to detect surface oxygen vacancies in oxide catalysts.^72,73^ As depicted in Fig. 2, this method uses the surface oxygen vacancy sites of the material to catalyze the decomposition of H_2_O_2_ in a pH 4 buffer. The liberated hydroxides subsequently deprotonate the amine groups in the 3,3′,5,5′-tetramethylbenzidine probe. This reaction changes the color from clear to turquoise and is quantified with an optical absorbance measurement. Although this method gives an indirect quantification of surface oxygen vacancies, it has the advantages of background subtraction and dynamically probing the vacancy sites accessible for OER in a liquid environment.

### 5.2 Bulk techniques

Electron paramagnetic resonance (EPR) is also frequently used for semi-quantitative oxygen vacancy characterization. Yet, only single unpaired electrons trapped at oxygen vacancies are directly detected at the common *g* ≈ 2.002 signal, which represents only a subset of the oxygen vacancy population.^74^ A rigorous quantification requires analysis of the catalysts valence state (cation reduction) and magnetic structure.^74–77^

Given accurate cation ratio constraints, hard XAS allows bulk oxygen vacancies to be calculated. First, the cation K-shell absorption edge energy is detected. Then, standards of known oxidation state are used to linearly correlate edge energies with the oxidation state. Finally, the oxygen content is determined by charge neutrality of the cation oxidation state. Fitting neutron diffraction data can give more precise information on oxygen vacancies; however, large amounts (≈4 g) and highly crystalline samples are usually required.^78^ XRD using a synchrotron source can be used for smaller masses, yet the technique is inherently less sensitive than neutron diffraction to light elements and their disorder.

Thermogravimetric analysis (TGA) can be used to directly measure the mass loss associated with the formation of oxygen vacancies; however, one should know the exact off-gassing species (H_2_O, CO_2_, O_2_) to make an accurate back calculation of the O content and origin. TGA coupled with in-line ICP-MS or ICP-OES can simultaneously detect the exhaust gas composition. Our experience suggests that a high purity sample is required to avoid ambiguous signals from incorporated/adsorbed solvents and trace byproducts.

Iodometric titration can give precise oxygen stoichiometry results, but the sample should be a single phase and ideally contain only one metal ion with changing oxidation state for accurate back calculation.^78,79^ A thorough understanding of the valence states of multimetallic oxides, especially Co and Fe, should be obtained with a complementary technique to avoid ambiguous titration results. Selective complexation can help isolate different ions in some cases.^80^

## 6 Summary and future perspectives

In Concept 1, we clarify that the effects of oxygen vacancies include shifting the electronic band alignments, adsorbing reaction intermediates, and initiating surface reconstruction. Cation vacancies can also affect the adsorption energies and surface reconstruction pathway *via* relative dissolution rates. A dynamic equilibrium between catalyst bulk, surface, and double layer is required for durability. In Concept 2, we establish that the effects of oxygen vacancies on a material's electronic properties and surface reconstruction are facet dependent. Combining doping and selective vacancy strategies is promising for activating CoOOH/NiOOH OER active surfaces. In Concept 3, we show that the precise introduction of oxygen vacancies can selectively change the dominant reaction coordinate of the OER between the AEM and the more active LOM. In Concept 4, we summarize and examine recent *operando* works on possible surface reconstruction pathways in Co-based OER catalysts. Oxygen vacancy-mediated adsorption of OH^−^ and H_2_O at low applied potentials is key for a highly active reconstructed CoOOH surface. However, high oxygen vacancy densities can initiate chemical or electrochemical active site quenching. In Concept 5, we highlight a remarkably common oxygen vacancy quantification XPS error (531 eV O 1s) and critically examine more accurate quantification techniques.

There is mounting evidence that oxygen vacancies, formed both *via* precatalyst modification methods and *in situ* metal dissolution, can determine the dominant surface reconstruction pathway and ultimate electrocatalyst activity. Fully understanding and influencing the surface reconstruction pathway of OER catalysts promises to increase the reconstructed active site density and activity. By continuing to combine *operando* surface characterization with rigorous oxygen vacancy quantification techniques, we expect that the vacancy thresholds for selectively steering the reaction mechanism and reconstruction pathway will be understood in the near future. Future work should focus on systematically introducing—and thoroughly quantifying— selected oxygen vacancy densities in a range of materials to observe trends in the mechanism steering thresholds. Selectively activating the LOER will enable escape from the universal scaling relationships limiting the activity of catalysts following the AEM.

## Author contributions

KC: conceptualization, investigation, visualization, writing – original draft; TJS: supervision, writing – review & editing; EF: conceptualization, funding acquisition, project administration, supervision, writing – review & editing.

## Conflicts of interest

There are no conflicts to declare.

## Data Availability

No primary research results, software or code have been included and no new data were generated or analyzed as part of this review.
